# Factors Associated with Mortality and Graft Failure in Liver Transplants: A Hierarchical Approach

**DOI:** 10.1371/journal.pone.0134874

**Published:** 2015-08-14

**Authors:** Luciana Haddad, Alex Jones Flores Cassenote, Wellington Andraus, Rodrigo Bronze de Martino, Neli Regina de Siqueira Ortega, Jair Minoro Abe, Luiz Augusto Carneiro D’Albuquerque

**Affiliations:** 1 Digestive Transplant Unit—Gastroenterology Department, São Paulo University, São Paulo, Brazil; 2 Postgraduate Program in Infectious and Parasitic Diseases, Faculty of Medicine, University of São Paulo, São Paulo, Brazil; 3 University of São Paulo, Faculty of Medicine, Center of Fuzzy Systems in Health, São Paulo, São Paulo, Brazil; 4 Institute for Advanced Studies, University of São Paulo, São Paulo, Brazil; UNIFESP Federal University of São Paulo, BRAZIL

## Abstract

**Background:**

Liver transplantation has received increased attention in the medical field since the 1980s following the introduction of new immunosuppressants and improved surgical techniques. Currently, transplantation is the treatment of choice for patients with end-stage liver disease, and it has been expanded for other indications. Liver transplantation outcomes depend on donor factors, operating conditions, and the disease stage of the recipient. A retrospective cohort was studied to identify mortality and graft failure rates and their associated factors. All adult liver transplants performed in the state of São Paulo, Brazil, between 2006 and 2012 were studied.

**Methods and Findings:**

A hierarchical Poisson multiple regression model was used to analyze factors related to mortality and graft failure in liver transplants. A total of 2,666 patients, 18 years or older, (1,482 males; 1,184 females) were investigated. Outcome variables included mortality and graft failure rates, which were grouped into a single binary variable called negative outcome rate. Additionally, donor clinical, laboratory, intensive care, and organ characteristics and recipient clinical data were analyzed. The mortality rate was 16.2 per 100 person-years (py) (95% CI: 15.1–17.3), and the graft failure rate was 1.8 per 100 py (95% CI: 1.5–2.2). Thus, the negative outcome rate was 18.0 per 100 py (95% CI: 16.9–19.2). The best risk model demonstrated that recipient creatinine ≥ 2.11 mg/dl [RR = 1.80 (95% CI: 1.56–2.08)], total bilirubin ≥ 2.11 mg/dl [RR = 1.48 (95% CI: 1.27–1.72)], Na^+^ ≥ 141.01 mg/dl [RR = 1.70 (95% CI: 1.47–1.97)], RNI ≥ 2.71 [RR = 1.64 (95% CI: 1.41–1.90)], body surface ≥ 1.98 [RR = 0.81 (95% CI: 0.68–0.97)] and donor age ≥ 54 years [RR = 1.28 (95% CI: 1.11–1.48)], male gender [RR = 1.19(95% CI: 1.03–1.37)], dobutamine use [RR = 0.54 (95% CI: 0.36–0.82)] and intubation ≥ 6 days [RR = 1.16 (95% CI: 1.10–1.34)] affected the negative outcome rate.

**Conclusions:**

The current study confirms that both donor and recipient characteristics must be considered in post-transplant outcomes and prognostic scores. Our data demonstrated that recipient characteristics have a greater impact on post-transplant outcomes than donor characteristics. This new concept makes liver transplant teams to rethink about the limits in a MELD allocation system, with many teams competing with each other. The results suggest that although we have some concerns about the donors features, the recipient factors were heaviest predictors for bad outcomes.

## Introduction

Liver transplantation has gained increased attention in the medical field since the 1980s due to the introduction of new immunosuppressants and improved surgical techniques. These factors have also improved outcomes, and a huge expansion in transplants and institutions performing this procedure has occurred. Currently, liver transplantation is the treatment of choice for patients with end-stage liver disease, and this treatment has been expanded to other indications as well.

Liver transplantation outcomes depend on donor factors, operating conditions, and recipient liver disease stage [1–3]. The DRI (Donor Risk Index) suggested by Feng *et al* is useful for calculating the liver graft risk based on donor variables [1]. The MELD (Model for End stage Liver Disease) score is the best predictor of waitlist mortality but has not been validated for measuring transplant survival rates because it only includes recipient data [4]. Other models, such as D-MELD, SOFT, and BAR scores, can improve fairness in the allocation of the limited organs available for transplantation [2,3,5]. Although these scores are useful, they have only been validated for American and European populations and transplant services. Previously, these tools had not been validated in developing countries, which have lower levels of donor care and longer wait lists.

The high rates of favorable outcomes in liver transplants have raised the hopes of patients with end-stage liver disease. However, the increased need for liver allografts has led to a broadened set of criteria for organ acceptability, increasing the risk of adverse outcomes.

In Brazil, 14,761 liver transplants were performed between 2002 and 2012. In 2013, 1,723 liver transplants were performed. However, this number is insufficient for a country with an expected annual requirement of 4,769 transplants [6]. São Paulo State is the most populous region in the country and was responsible for 37.6% of all liver transplants last year. The region has a very long waiting list, and organ shortage remains a critical problem within the transplant community. As a result, MELD system allocation was implemented in 2002 [6]. The current shortage of available organs has resulted in the use of expanded donor criteria to match donors with recipients with high MELD scores.

The aim of this study was to evaluate the factors associated with mortality and graft failure in all adult transplants performed in the state of São Paulo, Brazil, between 2006 and 2012.

## Methods

### Study design, variables, outcome definitions and ethical considerations

**Fig 1:**
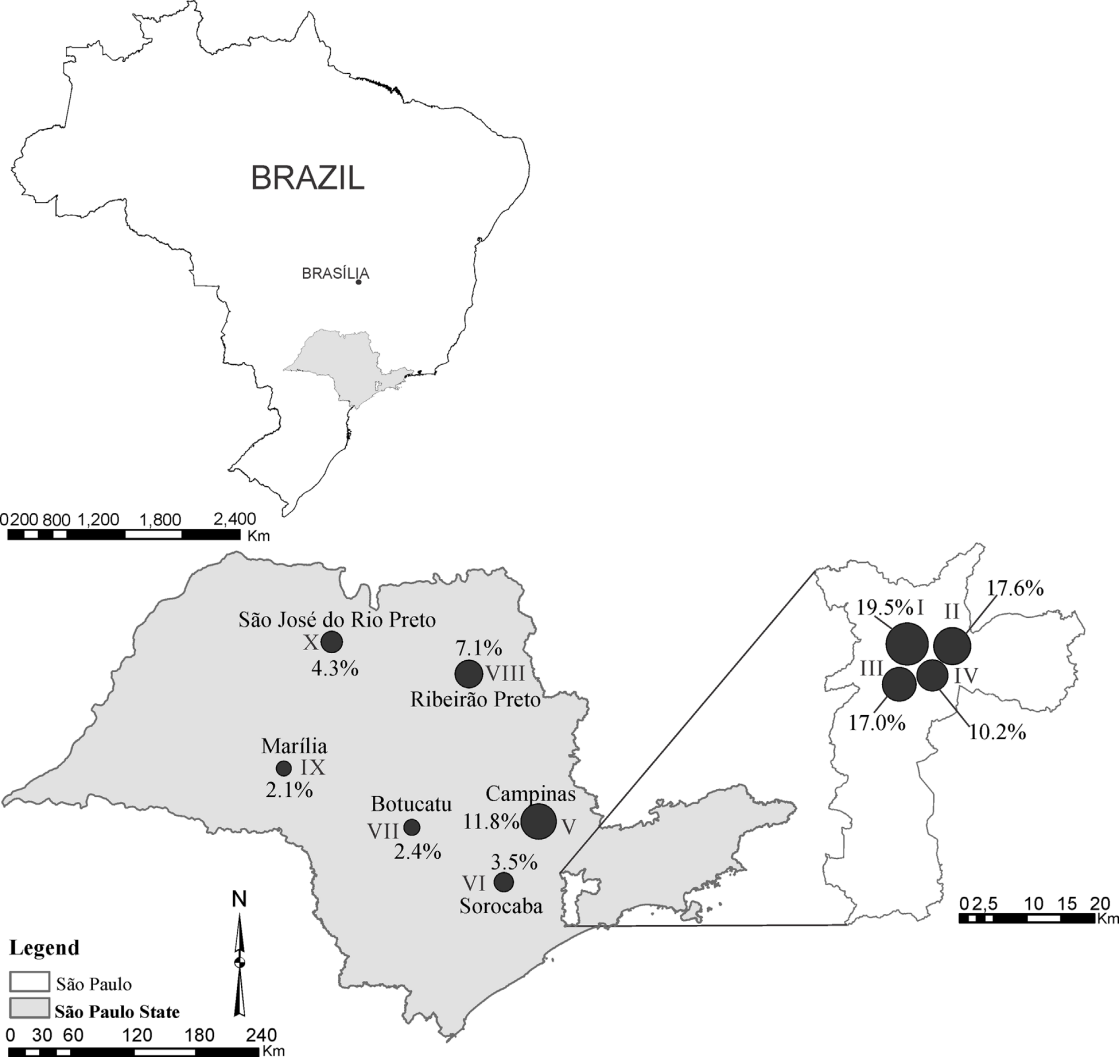
Relative distribution of livers according to organ collection center between 2006 and 2012 in São Paulo, Brazil. In total, 4.5% (122) of the livers came from outside São Paulo.

This study evaluated a retrospective cohort of all adult patients (18 years or older) in São Paulo State, Brazil, who received liver transplants between 01/01/2006 and 08/30/2012 (Fig 1); combined transplants (liver—kidney combination) and retransplantation cases were excluded from this study. The Secretary of Health of São Paulo State provided the database for this study.

Individual donor and recipient variables available in the database were grouped as follows:

1. Donor clinical data: age, body mass index—BMI—(mass [kg]/height [m]^2^), body surface ((0.007184*(height [cm])*0.725)*(mass [kg])0.425), gender and cause of death (stroke, cranioencephalic trauma and other);
2. Donor laboratory data: serum Na^+^ (sodium) (mg/dl), serum GOT (glutamic-oxaloacetic transaminase) (mg/dl), serum GPT (glutamic-pyruvic transaminase) (mg/dl), alkaline-phosphatase (mg/dl), glutamyl-transferase (mg/dl), total bilirubin (mg/dl), and creatinine (mg/dl);
3. Donor intensive care data: intensive care days; intubation days; use of dopamine, dobutamine, and noradrenaline;
4. Organ characteristics: cold ischemia time (hours) and warm ischemia time (hours);
5. Recipient clinical data: age (year), body mass index (mass [kg]/height [m]^2^) and body surface ((0.007184*(height [cm])*0.725)*(mass [kg])0.425);
6. Recipient laboratory data: creatinine (mg/dl), total bilirubin (mg/dl), serum international normalized ratio (INR), serum Na^+^ (mg/dl);
7. Other features: recipient MELD Score (0.957*ln[serum creatinine]+0.378*ln[serum bilirubin]+1.120*ln[INR]+0.643)*10 (if hemodialysis, the value for creatinine is automatically set to 4.0), outcome (death and graft failure) and outcome causes (arterial complication, venous complication, bacterial infection, fungal infection, viral infection, metabolic complication, neoplasia and unspecified).

The origin of the organ, according to the organ collection center (OCC), was the geographical context variable available in the database. The São Paulo Transplant Center (SPTC) coordinates 10 OCCs, including 4 in São Paulo City (I, II, III and IV) and 6 in countryside states (V, VI, VII, VIII, IX and X) (Fig 1).

Outcome measures included the mortality rate and graft failure rate, grouped into a single binary variable (yes/no) called the negative outcome (NO) rate. Continuous quantitative independent variables were binarized by percentile rank, with the third quartile serving as the cutoff reference.

The study was reviewed and approved by the Research Ethics Committee of the Clinics Hospital, São Paulo University Medical School (Protocol Number #9949–13), in accordance with Brazilian and international regulations for research with human subjects. The institutional review board (IRB) waived the individual requirement for written informed consent, and they requested written consent from the director of SPTC because this was a retrospective study. The board also requested confidentiality of the individuals’ data, which was ensured at all stages of the project. The following information was de-identified: name, address, zip code, registration, donor's name and donor registry. None of the transplant donors were from a vulnerable population and all donors or next of kin provided written informed consent that was freely given.

### Hierarchical framework modeling

A hierarchical multiple regression model was considered to study factors related to mortality and graft failure in liver transplants. This analysis is generally used to explain the relationship between variables in models with a set of empirical propositions already indicating the relationship strength and direction between predictors and outcomes. The order of predictor entry in the regression equation was defined by the researcher based on a pre-established conceptual framework [7].

Building a conceptual framework requires knowledge of the biological and temporal determination affecting outcomes [8]. Using the dataset, a team of 8 experts grouped relevant variables into 6 distinct analytical blocks. Then, using a score system of 0 to 5 per variable, the experts described a hierarchical relationship between predictors and outcomes. Surgeons from liver transplantation teams with at least two years of experience in centers performing more than 50 transplants/year were considered experts.

**Fig 2:**
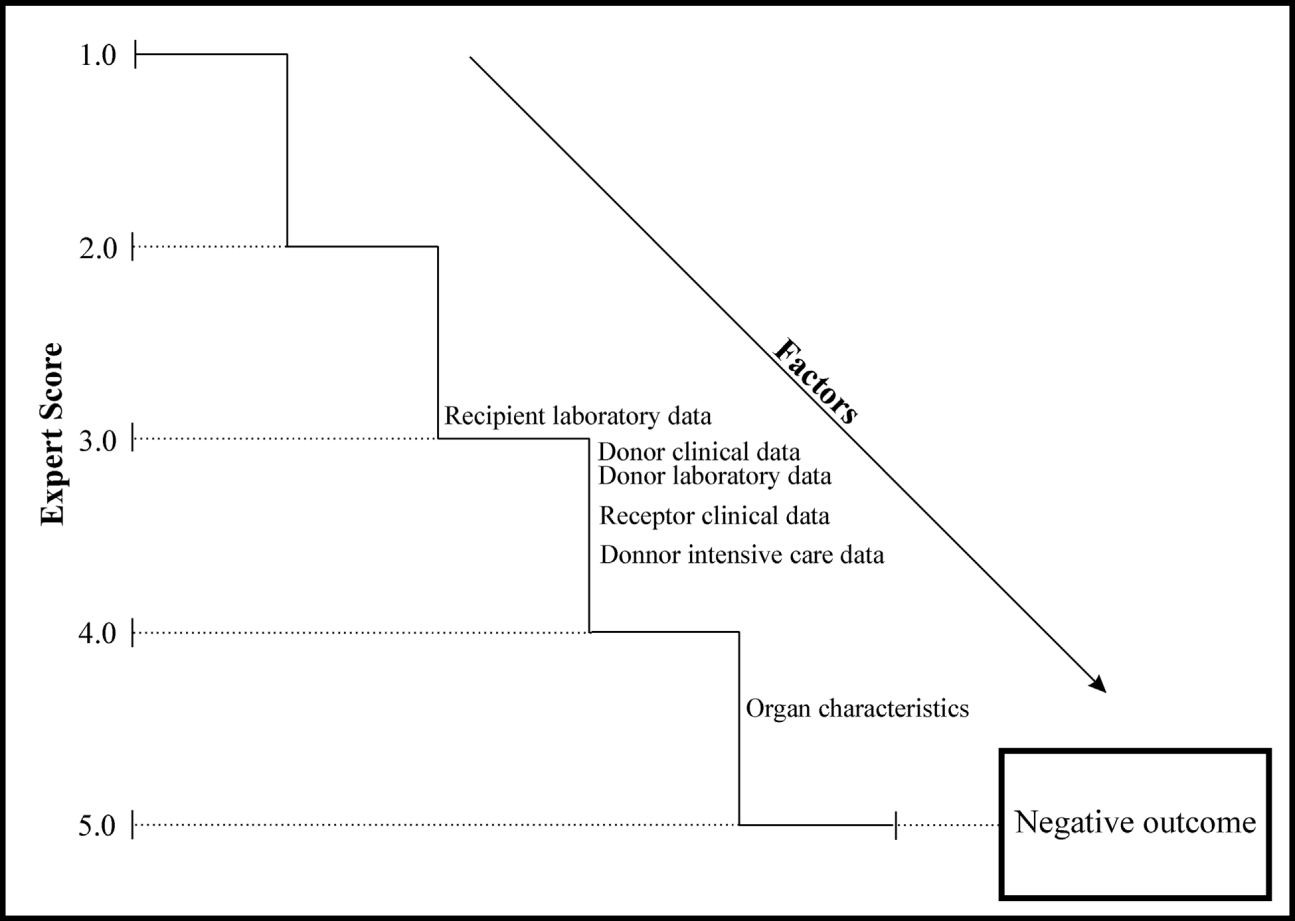
Theoretical framework used to assess factors associated with mortality and graft failure in liver transplants in São Paulo between 2006 and 2012.

The position of a specific set of variables regarding the outcome was based on the arithmetic mean of the scoring system, named the expert score (ES). Organ characteristics data were the most proximal terms (4 to 5 points). Medial terms (3 to 4.99 points) included donor clinical data, donor laboratory data, donor intensive care data and recipient clinical data. The distal terms (1 to 2.99 points) included recipient laboratory data (Fig 2).

### Statistical analysis

Data are shown as absolute frequency, means with standard deviations (SD), proportion with confidence intervals (95% CI), and medians with interquartile ranges (IQRs) unless otherwise stated [9,10]. Crude incidence rates (mortality, graft failure, and NO) were defined as the number of cases per 100 person-years with a corresponding 95% CI and assuming a Poisson distribution [11,12].

To estimate survival time until death, graft failures and negative outcomes were used based on the Kaplan-Meier method [10,13]. To study influences on incidence rate, bivariate and multiple analysis using generalized linear models (GLMs) with a Poisson distribution and the “log” link function were fitted. Time was used as an offset variable, enabling relative risk (RR) to be described [14,15]. The adjustment of different models was verified by indicators of residual deviance and the Akaike information criterion (AIC). An ANOVA was used to verify the equality hypothesis among the different models [12,16].

To improve the different models, predictor variables were tested for collinearity with the variance inflation factor (VIF) as well as for the presence of influential values. Model accuracy was evaluated using a cross-validation system [13,15].

The database was exported to the Statistical Package for the Social Sciences (SPSS) 22 for Windows (International Business Machines Corp, New York, USA) and R-GUI version 3.0.2 (http://www.r-project.org/) for statistical treatments. All the significance levels were set to p<0.05.

## Results

The clinical and laboratory profiles of the 2,666 patients enrolled between 2006 and 2012 are shown in Table 1. The donors were men in 55.6% of cases (male/female ratio 1.25:1). The mean age at enrollment was 40.9 years (SD 15.8 years), and the mean BMI was 25.2 (SD 3.8). Cerebral vascular accident (CVA) was the largest cause of death, at 59.4% (CI 95% 57.7–61.1). Among the recipients, the mean age was 49.6 years (SD 13.7 years) and the mean BMI was 26.0 (SD 4.7); the mean MELD score was 23.5 (SD 11.2), with 25% of patients scoring above 32.

**Table 1:** Descriptive statistics: mean with standard deviation (SD) or proportion (%) with 95% confidence interval (95% CI), median with interquartile range (IQR), and expert score (ES) of variables from donor, organ and recipient of liver transplants in São Paulo, Brazil.

	n	Mean (SD) or % (CI 95%)	Median (IQR)	ES^f^	Mean of ES^g^
**Donor clinical data**					
Age (year)	2666	40.9 (15.8)	43.0 (28.0–53.0)	4.6	3.1
Body mass index	2666	25.2 (3.8)	24.8 (23.1–27.3)	4.0	
Body surface	2666	1.8 (0.2)	1.9 (1.7–1.9)	1.9	
Gender				1.2	
Female	1184	44.4 (42.6–46.4)			
Male	1482	55.6 (53.6–57.4)			
Cause of death				3.7	
CVA^a^	1583	59.4 (57.7–61.1)			
TBI^b^	1008	37.8 (36.0–39.6)			
Another	75	2.8 (2.2–3.4)			
**Donor laboratory data**					
NA+ (mg/dl)	2666	156.7 (14.8)	156.0 (146.0–166.0)	2.9	3.2
GOT^c^ (mg/dl)	2666	102.9 (227.6)	52.0 (31.0–97.3)	3.4	
GPT^d^ (mg/dl)	2666	75.6 (172.4)	39.0 (24.0–72.0)	3.5	
Alkaline-phosphatase (mg/dl)	2643	127.2 (93.6)	96.0 (67.0–159.0)	2.9	
Glutamyl-transferase (mg/dl)	2647	89.2 (106.9)	47.0 (23.0–110.0)	3.7	
Total bilirubin (mg/dl)	2666	0.7 (0.8)	0.5 (0.3–0.8)	3.7	
Creatinine (mg/dl)	2666	1.7 (1.5)	1.3 (0.9–2.0)	2.7	
**Donor intensive care data**					
Intensive care days	2661	5.2 (4.7)	4.0 (2.0–7.0)	4.1	3.6
Intubation days	2666	5.0 (3.9)	4.0 (2.0–6.0)	3.5	
Dopamine use				3.0	
No	2316	86.9 (85.6–88.2)			
Yes	350	13.1 (11.8–14.4)			
Dobutamine use				3.0	
No	2564	96.2 (95.5–96.9)			
Yes	102	3.8 (3.1–4.5)			
Noradrenaline use				4.2	
No	470	17.6 (16.2–19.2)			
Yes	2196	82.4 (80.8–83.8)			
**Organ characteristics**					
Cold ischemia time (hours)	2666	8.0 (2.5)	7.8 (6.1–9.5)	4.5	4.4
Warm Ischemia time (hours)	2666	0.8 (0.3)	0.8 (0.7–0.9)	4.4	
**Recipient clinical data**					
Age (year)	2666	49.6 (13.7)	53.0 (43.0–59.0)	4.1	3.4
Body mass index	2665	26.0 (4.7)	25.4 (22.8–28.7)	3.2	
Body surface	2665	1.8 (0.2)	1.8 (1.7–2.0)	2.8	
**Recipient laboratory data**					
Creatinine (mg/dl)	2556	1.7 (1.4)	1.1 (0.8–2.1)	3.5	2.9
Total bilirubin (mg/dl)	2556	9.5 (11.5)	4.6 (1.8–12.7)	3.	
INR	2556	2.4 (1.9)	1.9 (1.4–2.7)	2.8	
NA+ (mg/dl)	2556	137.7 (6.2)	138.0 (134.0–141.0)	2.3	
**Other features**					
Meld	2666	23.5 (11.2)	23.0 (14.0–32.0)		
**Outcome**					
Death	897	33.6 (31.8–35.5)			
Graft failure	100	3.8 (3.0–4.4)			
Outcome causes					
Arterial complication	56	6.7 (5.1–8.2)			
Venous complication	15	1.8 (1.0–2.7)			
Bacterial infection	251	30.0 (27.0–33.3)			
Fungal infection	10	1.2 (0.5–2.0)			
Viral infection	10	1.2 (0.5–2.0)			
Metabolic complication	84	10.0 (8.0–12.1)			
Neoplasia	33	3.9 (2.7–5.4)			
Unspecified	36	4.3 (2.9–5.7)			
Another^e^	342	40.9 (37.6–44.1)			

^a^ Cerebral vascular accident

^b^ Traumatic brain injury

^c^ Glutamic-oxaloacetic transaminase

^d^ Glutamic-pyruvic transaminase

^e^ Rejection, cardiologic causes, trauma, etc

^f^ Expert score per variable (5 is closest to outcome)

^g^ Mean of expert score per block of variables (5 is closest to outcome)

**Fig 3:**
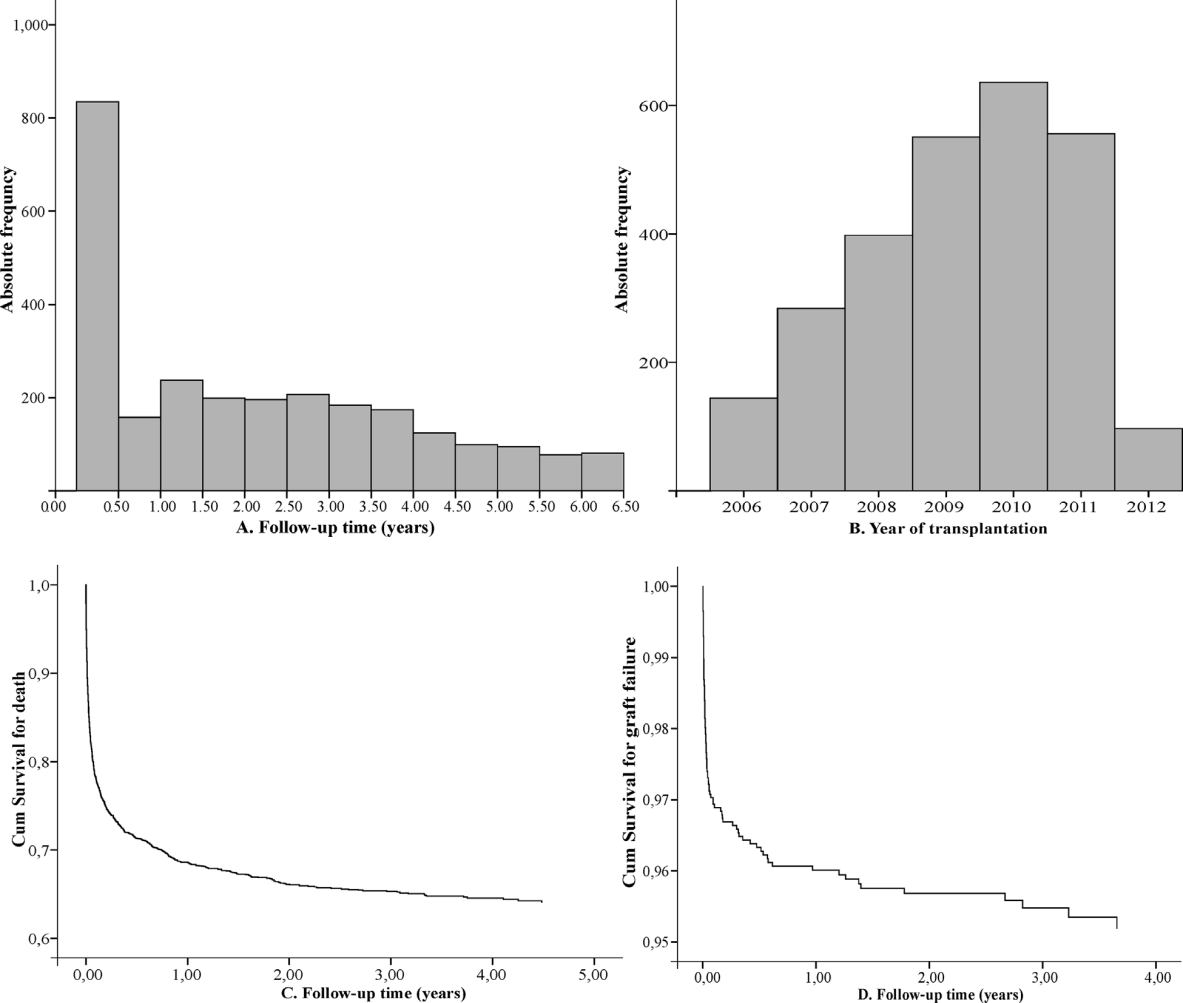
Characterization of follow-up time, year of liver transplantation, cumulative survival before death, and graft failure in the set of evaluated patients.

The total follow-up time was 5,526.09 person-years (py), with a mean of 2.07 (SD 1.87) per person. The median follow-up duration was 1.75 years (IQR 0.11–3.44 years; maximum 6.48 years). The transplantation midpoint time was 2009 (SD 1.55). Thus, most transplants were concentrated between 2008 and 2010, as shown in Fig 3A and 3B.

We recorded 897 (33.6%) deaths and 100 (3.8%) cases of graft failure (Table 1). Fig 3C and 3D present the survival curves for the two outcomes. The survival rate after liver transplantation was 68.5% in the first year vs. 65.5% in the third y (the mean was 4.29 years [95% CI: 4.17–4.40]). Graft failure was 96.0% in the first year vs. 95.5% in the third year (the mean was 6.19 years [95% CI: 6.13–6.24]). The mortality rate was 16.2 per 100 py (95% CI: 15.1–17.3), and the graft failure rate was 1.8 per 100 py (95% CI: 1.5–2.2). Thus, the NO rate was 18.0 per 100 py (95% CI: 16.9–19.2). All outcome rates were homogeneous among CCOs (Table 2).

**Table 2:** Mortality rate, graft failure rate and negative outcome (NO) rate with a 95% confidence interval (95% CI) according to OCC in São Paulo, Brazil.

OCC	Livers	Person-Years	Death	Mortality rate (95% CI)^a^ ^,^ ^b^	Graft Failure	Graft Failure Rate (95% CI) ^a^ ^,^ ^b^	Negative Outcome^c^	Negative Outcome Rate (95% CI) ^a^ ^,^ ^b^
I	520	1131.76	159	14.0 (11.9–16.4)	24	2.1 (1.3–3.1)	183	16.1 (13.9–18.6)
II	468	923.73	162	17.5 (14.9–20.4)	20	2.1 (1.3–3.3)	182	19.9 (17.1–23.0)
III	452	914.43	153	16.7 (14.1–19.6)	15	1.6 (0.9–2.7)	168	18.3 (15.6–21.3)
IV	272	571.46	95	16.6 (13.4–20.3)	6	1.0 (0.3–2.2)	101	17.6 (14.3–21.4)
V	314	659.20	102	15.4 (12.6–18.7)	18	2.7 (1.6–4.3)	120	18.2 (15.0–21.7)
VI	93	219.12	29	13.2 (8.8–19.0)	2	0.9 (0.1–3.2)	31	14.1 (9.6–20.0)
VII	64	138.73	22	15.8 (9.9–24.0)	2	1.4 (0.1–5.2)	24	17.2 (11.0–25.7)
VIII	190	358.60	74	20.6 (16.2–25.9)	6	1.6 (0.6–3.6)	80	22.3 (17.3–17.7)
IX	56	102.31	20	19.5 (11.9–30.1)	1	0.9 (0.2–5.4)	21	20.5 (12.7–31.3)
X	115	254.78	39	15.3 (10.8–20.9)	4	1.5 (0.4–4.0)	43	16.8 (12.8–16.7)
TOTAL	2544	5274.12	855	16.2 (15.1–17.3)	98	1.8 (1.5–2.2)	953	18.0 (16.9–19.2)

- 122 livers came from outside of São Paulo (251.97 py): mortality rate: 16.6 (12.0–22.5); graft failure rate: 0.7 (0.1–2.8); NO rate: 17.4 (12.6–23.4)

^a^ p-value = 0.980 (random effect)

^b^ Considering the sum of total deaths and total graft failure

^c^ per 100 person-years (py)

Crude analysis showed that the following donor factors were significantly associated with a higher NO rate: age above 53 years, male gender, intubation time longer than 5 days, dobutamine use and total bilirubin lower than 0.81 mg/dl. Recipient characteristics contributing to a higher NO rate included creatinine levels higher than 2.10 mg/dl, total bilirubin higher than 2.11 mg/dl, INR higher than 2.70, Na^+^ higher than 141 mg/dl and body surface lower than 1.98 (Table 3).

**Table 3:** Hierarchical model used to study mortality rate and graft failure with crude and adjusted relative risk and 95% confidence interval (95% CI) in São Paulo, Brazil.

	Negative Outcome		Crude analysis		Model per block		EM^h^	Model I		Modal II		Model III		Model IV		Model V	
	n (%)	in^g^	p-value	RR (95% CI)	p-value	RR (95% CI)		p-value	RR (95% CI)	p-value	RR (95% CI)	p-value	RR (95% CI)	p-value	RR (95% CI)	p-value	RR (95% CI)
**Recipient laboratory data**																	
**Creatinine (mg/dl)**																	
0.10 to 2.10	665 (34.4)	152.77															
2.11 to 12.40	311 (49.5)	299.46	<0.001	1.96 (1.71–2.24)	<0.001	1.83 (1.59–2.10)		<0.001	1.81 (1.57–2.07)	<0.001	1.80 (1.56–2.08)	<0.001	1.80 (1.56–2.08)	<0.001	1.80 (1.56–2.08)	<0.001	1.80 (1.56–2.07)
Overall	976 (38.1)	181.03															
**Total bilirubin (mg/dl)**																	
0.20 to 2.10	653 (34)	153.2															
2.11 to 75.00	323 (50.6)	286.09	<0.001	1.87 (1.63–2.13)	<0.001	1.49 (1.29–1.73)		<0.001	1.49 (1.29–1.73)	<0.001	1.50 (1.29–1.74)	<0.001	1.50 (1.29–1.74)	<0.001	1.48 (1.27–1.72)	<0.001	1.48 (1.27–1.73)
Overall	976 (38.1)	181.03															
**INR**																	
0.88 to 2.70	657 (33.9)	150.44															
2.71 to 15.00	319 (51.3)	307.36	<0.001	2.01 (1.75–2.29)	<0.001	1.61 (1.39–1.86)		<0.001	1.62 (1.4–1.88)	<0.001	1.61 (1.39–1.87)	<0.001	1.64 (1.40–1.90)	<0.001	1.64 (1.41–1.90)	<0.001	1.64 (1.41–1.90)
Overall	976 (38.1)	181.03															
**NA+ (mg/dl)**																	
109.00 to 141.00	711 (36.1)	163.77															
141.01 to 170.00	265 (44.9)	252.39	<0.001	1.54 (1.34–1.77)	<0.001	1.69 (1.47–1.95)		<0.001	1.68 (1.46–1.94)	<0.001	1.69 (1.47–1.95)	<0.001	1.70 (1.48–1.96)	<0.001	1.70 (1.47–1.97)	<0.001	1.69 (1.46–1.95)
Overall	976 (38.1)	181.03															
**Donor clinical data**																	
**Age (years)**																	
18 to 53	739 (35.9)	169.7															
54 to 82	258 (42.4)	220.05	<0.001	1.30 (1.13–1.49)	<0.001	1.30 (1.13–1.50)		<0.001	1.29 (1.12–1.49)	<0.001	1.31 (1.10–1.51)	<0.001	1.28 (1.11–1.48)	<0.001	1.28 (1.11–1.49)	0.001	1.27 (1.10–1.47)
Overall	997 (37.3)	180.38															
**Body mass index** ^**a**^																	
12.00 to 27.34	808 (36.9)	193.01															
27.35 to 58.00	189 (39.4)	140.95	0.08	1.13 (0.98–1.3)													
Overall	997 (37.3)	180.38															
**Body surface**																	
0.552 to 1.941	732 (36.5)	172.69															
1.942 to 2.571	265 (39.2)	205.68	0.05	1.16 (1.00–1.36)	0.36	1.08 (0.92–1.27)		0.900	0.95 (0.76–1.22)	0.901	0.98 (0.79–1.23)	0.815	0.99 (0.83–1.17)	0.943	0.98 (0.82–1.19)	0.945	0.98 (0.83–1.17)
Overall	997 (37.2)	180.38															
**Gender**																	
Female	420 (35.4)	165.35															
Male	577 (38.9)	193.15	0.015	1.17 (1.03–1.32)	0.044	1.15 (1.00–1.32)		0.010	1.2 (1.05–1.38)	0.01	1.2 (1.05–1.38)	0.013	1.2 (1.04–1.38)	0.016	1.19 (1.03–1.37)	0.018	1.19 (1.03–1.37)
Overall	997 (37.3)	180.38															
**Cause of death**																	
CVA^b^	599 (37.8)	185.87															
TBI^c^	371 (36.8)	174.31	0.331	1.28 (0.61–1.32)	0.422	1.11 (0.55–1.43)		0.420	1.18 (0.53–1.47)	0.423	1.18 (0.53–1.47)	0.426	1.17 (0.52–1.49)	0.429	1.20 (0.50–1.51)	0.428	1.29 (0.50–1.51)
Another	27 (36.0)	153.13	0.325	1.22 (0.81–1.28)	0.528	1.08 (0.68–1.55)		0.536	1.11 (0.69–1.59)	0.539	1.11 (0.69–1.59)	0.54	1.10 (0.66–1.58)	0.542	1.12 (0.65–1.60)	0.541	1.11 (0.65–1.60)
Overall	997 (37.3)	180.38															
**Donor laboratory data**																	
**NA+ (mg/dl)**																	
105,00 to 166,00	743 (37.1)	179.23															
167,00 to 200,00	254 (38.1)	183.81	0.729	1.03 (0.89–1.18)	0.935	1.00 (0.87–1.16)				0.816	1.02 (0.88–1.18)	0.938	1.00 (0.86–1.17)	0.962	1.00 (0.86–1.16)	0.996	1.00 (0.86–1.15)
Overall	997 (37.3)	180.38															
**GOT (mg/dl)** ^**d**^																	
5.00 a 97.25	751 (37.5)	182.32															
97.26 a 5480.00	246 (36.9)	174.69	0.561	0.96 (0.83–1.11)	0.63	0.96 (0.83–1.12)				0.891	0.99 (0.85–1.15)	0.856	0.99 (0.85–1.15)	0.903	0.99 (0.85–1.15)	0.903	0.99 (0.85–1.15)
Overall	997 (37.3)	180.38															
**GPT (mg/dl)** ^**a**^ ^,^ ^**e**^																	
5.00 to 72.00	757 (37.6)	180.98															
72.01 to 4521.00	240 (36.4)	178.5	0.852	0.99 (0.85–1.14)													
Overall	997 (37.3)	180.38															
**Alkaline-phosphatase (mg/dl)**																	
5.00 to 159.00	746 (37.5)	181.79															
159.01 to 1000.00	246 (37.5)	179.86	0.884	0.99 (0.86–1.14)	0.841	0.99 (0.85–1.14)				0.691	0.97 (0.83–1.13)	0.642	0.96 (0.83–1.12)	0.545	0.95 (0.82–1.11)	0.536	0.95 (0.82–1.11)
Overall	992 (37.5)	181.31															
**Glutamyl-transferase (mg/dl)**																	
3.00 to 110.00	739 (37.1)	179.7															
110.01 to 600.00	256 (38.9)	187.35	0.565	1.04 (0.90–1.20)	0.392	1.07 (0.92–1.13)				0.217	1.10 (0.95–1.28)	0.195	1.10 (0.95–1.28)	0.2	1.10 (0.95–1.28)	0.194	1.11 (0.95–1.29)
Overall	995 (37.5)	181.61															
**Total bilirubin (mg/dl)**																	
0.01 to 0.80	781 (38.3)	187.41															
0.81 to 17.00	216 (34.4)	158.83	0.031	0.85 (0.73–0.99)	0.028	0.85 (0.73–0.98)				0.054	0.86 (0.73–1.00)	0.067	0.87 (0.74–1.01)	0.084	0.87 (0.75–1.02)	0.095	0.88 (0.75–1.02)
Overall	997 (37.3)	180.38															
**Creatinine (mg/dl)**																	
0.100 to 2.000	761 (37.6)	181.91															
2.001 to 15.000	236 (36.5)	175.6	0.635	0.97 (0.83–1.12)	0.861	0.94 (0.85–1.15)				0.919	0.99 (0.85–1.15)	0.784	0.98 (0.84–1.14)	0.895	0.99 (0.85–1.15)	0.95	0.99 (0.85–1.15)
Overall	997 (37.3)	180.38															
**Donor intensive care data**																	
**Intensive care days** ^**a**^																	
0 to 7	775 (36.8)	176.6															
8 to 90	221 (39.6)	195.97	0.172	1.11 (0.96–1.29)													
Overall	996 (37.4)	180.56															
**Intubation days**																	
0 to 5	736 (36.5)	173.58															
6 to 30	261 (39.8)	202.76	0.031	1.17 (1.02–1.35)	0.028	1.17 (1.02–1.35)						0.049	1.16 (1.05–1.34)	0.045	1.16 (1.10–1.34)	0.048	1.16 (1.10–1.34)
Overall	997 (37.3)	180.38															
**Dopamine use**																	
No	863 (37.2)	178.24															
Yes	134 (38.2)	195.51	0.319	1.10 (0.91–1.32)	0.124	1.17 (0.96–1.42)						0.196	1.14 (0.93–1.4)	0.23	1.13 (0.92–1.38)	0.272	1.12 (0.92–1.38)
Overall	997 (37.3)	180.38															
**Dobutamine use**																	
No	969 (37.7)	183.36															
Yes	28 (27.4)	115.42	0.016	0.63 (0.43–0.92)	0.01	0.61 (0.42–0.89)						0.004	0.54 (0.36–0.82)	0.003	0.54 (0.36–0.82)	0.003	0.54 (0.36–0.82)
Overall	997 (37.3)	180.38															
**Noradrenaline use**																	
No	173 (36.8)	181.08															
Yes	824 (37.5)	180.23	0.955	0.99 (0.84–1.17)	0.753	1.03 (0.86–1.23)						0.867	0.98 (0.82–1.18)	0.973	0.98 (0.82–1.19)	0.988	0.98 (0.82–1.19)
Overall	997 (37.3)	180.38															
**Recipient clinical data**																	
**Age (years)**																	
18 to 53	749 (38.6)	181.75															
54 to 82	248 (34.1)	176.35	0.681	0.97 (0.84–1.12)	0.573	0.96 (0.83–1.11)								0.967	0.99 (0.85–1.16)	0.858	0.99 (0.85–1.15)
Overall	997 (37.3)	180.38															
**Body mass index** ^**a**^																	
12.00 to 28.73	749 (37.6)	182.24															
27.74 to 46.00	248 (36.5)	175.32	0.597														
Overall	997 (37.4)	180.47															
**Body surface**																	
0.750 to 1.97	770 (38.3)	187.07															
1.98 to 2.70	227 (34.6)	161.17	0.048	0.86 (0.74–1.00)	0.0402	0.84 (0.7–0.99)								0.021	0.81 (0.68–0.97)	0.018	0.81 (0.68–0.96)
Overall	997 (37.4)	180.47															
**Organ characteristics**																	
**Cold ischemia time (hours)**																	
0 to 9.50	756 (37.7)	182.18															
9.60 to 20.00	241 (36.4)	174.96	0.585	0.96 (0.83–1.11)	0.601	0.96 (0.83–1.11)										0.328	0.93 (0.8–1.08)
Overall	997 (37.3)	180.38															
**Warm Ischemia time (hours)**																	
0 to 0.92	776 (37.6)	181.27														0.933	0.99 (0.85–1.15)
0.93 to 4.00	221 (36.4)	177.32	0.773	0.98 (0.84–1.14)	0.81	0.98 (0.84–1.14)											
Overall	997 (37.3)	180.38															
**Residual deviance**							14,759	8,420		8,426		8,405		8,399		8,408	
**AIC** ^**f**^							16,753	10,351		10,358		10,351		10,347		10,358	
**p-value (model I)**							<0.001										
**p-value (model II)**							<0.001	0.514									
**p-value (model III)**							<0.001	0.026		0.004							
**p-value (model IV)**							<0.001	0.014		0.003		0.097					
**p-value (model V)**							<0.001	0.084		0.035		0.837		0.027			

^a^ Variables not analyzed in multiple models because presented collinearity

^b^ Cerebral vascular accident

^c^ Traumatic brain injury

^d^ Glutamic-oxaloacetic transaminase

^e^ Glutamic-pyruvic transaminase

^f^ Akaike's information criterion (AIC)

^g^ Incidence per 100 persons-years

^h^ Empty model

A collinearity effect was observed in four variable pairs, including donor BMI vs. body surface, donor GPT vs. GOT, donor intensive care days vs. intubation days and recipient BMI vs. body surface. Thus, donor BMI, GPT, intensive care days and recipient BMI were not considered in subsequent models. No changes in the effects of significantly associated factors were evident from crude analysis (Table 3).

The best model, with the lowest residual deviance (8,399) and AIC (10,351) values, was IV; this model explained a greater amount of variance compared with an empty model (p-value<0.001), model I (p-value = 0.014), model II (p-value = 0.014) and model V (p-value = 0.027). However, it was not significantly better than model III (p-value = 0.097). After adjustment in the Poisson multiple regression model, most analyzed variables maintained or improved (donor body surface and dobutamine use) their statistical significance and effect. Only donor total bilirubin was lower than 0.81 mg/dl and exhibited no significant effect after the adjustment (Table 3).

## Discussion

The MELD score was implemented as an allocation system for liver transplants in the USA in February 2002 [17]. This model was implemented in Brazil in 2006 and has been validated for predicting mortality among patients on the waiting list [18]. However, the ability of the MELD score to predict post-transplant survival remains controversial. Brazil is a huge country and Sao Paulo is the most populous and developed region, receiving patients from the others states of the country. This region does about half of all transplants in the country, and the database used in this study is the country's largest. The data indicate the long waiting list time and the high MELD score of transplanted patients in this region in Brazil [2].

The post-transplant survival (Fig 3c) in this cohort (68.5%) is substantially lower, especially in the first year, than European (87% in first year) [19] and North American rates (85% in first year) [19,20]. However, similar results were found in Germany, where the MELD system was also introduced in 2006. Germany experienced a decreased mortality of patients on the waiting list after implementing the new allocation policy but also a decrease in the 1-year survival post-liver transplant from 90% to less than 80% as well as a continuous deterioration of the organ quality over the past 10 to 15 years [21,22].

Although liver transplant waiting list mortality is closely correlated with MELD score, and these advanced cirrhotic patients urgently require transplantation, extended criteria grafts in very ill patients can lead to worse post-transplant outcomes. However, survival benefit studies show that these patients benefit more from this procedure, even with some risk of a mismatch [23–25]. This dilemma has led to a search for prognostic risk factors and scores that minimize the side effects of such mismatches.

**Fig 4:**
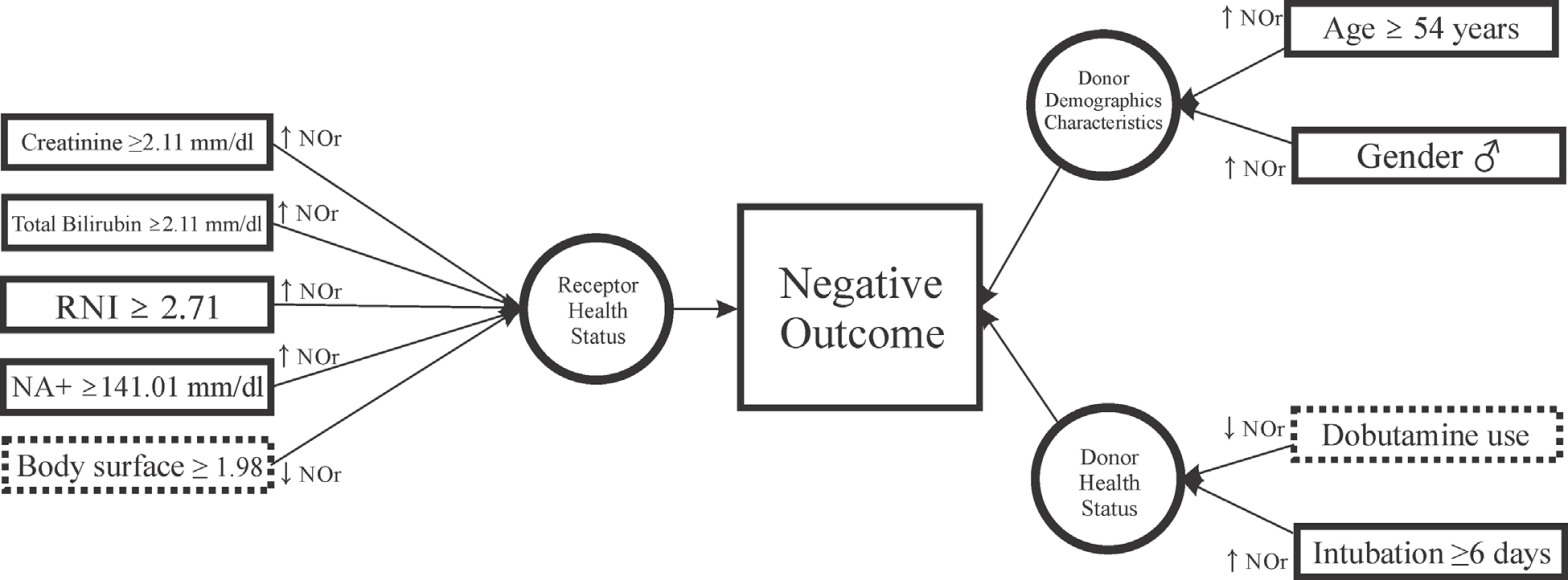
Final simplified framework of associated factors for mortality and graft failure in liver transplants in São Paulo between 2006 and 2012.

Fig 4 summarizes the best risk model (IV model) presented in Table 3. Recipient factors had a strong impact on the outcomes, which seem to reflect the health status of the recipient. In contrast, donor factors had a lesser impact upon the outcomes, although demographic characteristics of these donors, as well as their health status, were important.

Recipient creatinine, bilirubin and prothrombin time are biochemical markers of liver and kidney function and were shown to be much more relevant than any other donor characteristics. These markers are part of the MELD calculation, a score that is associated with short-term survival in patients with terminal hepatic insufficiency [4]. A high MELD score often indicates a negative outcome after liver transplantation by accurately reflecting disease severity [2,5,26–29].

Another recipient factor associated with better outcomes in our study was the body surface of the donor above 1.98. This finding is likely related to better nutrition in these patient groups. In a situation with a high percentage of critically ill patients, malnutrition is responsible for raising morbidity and mortality indices [30].

These results demonstrated that scores based only on donor data, such as the DRI (donor risk index) [1] and ECD score (extended-criteria donor score) [22], are not universally effective predictors of recipient success.

Other studies have evaluated recipient data [31] or both donor and recipient data [5,32]. However, these studies investigated patients from the USA or Europe, and outcome predictor scores from their results require validation worldwide. Regional differences can change predictor factors, as demonstrated here. For example, Feng et al. [1] identified eight donor risk factors related to transplant failure and developed the donor risk index score (DRI). One of the factors used in this score is race, but in countries such as Brazil, race is very difficult to determine because of historical miscegenation.

Regarding donor gender, some authors have reported an increased risk of graft loss as high as 20% when female donor organs have been transplanted into male recipients. However, others did not find such differences [33]. Other studies considering donor gender failed to find relations with prognostic variables [1,2,34]. In our study, recipients who received liver from male donors experienced more negative outcomes and we speculate that this result may be due to the association between gender donor with donor medical and/or behavioral conditions, in other words, confounding effect of variables, which were not measured. In this case, even without a specific reference of this association is crucial that this variable (gender) remains in the model to consider the effect of others variables in the multiple regression. Our study also found a 30% greater negative outcome rate in donors over 54 years old. However, the aging population and limited supply of donors are associated with a risk that should be considered in combination with other factors. Donor age is a predictor of hepatic transplantation outcome according to a number of studies [1,35].

Donor maintenance in many cases is not ideal in Brazil. Some hospitals do not have enough ICU beds for every patient and experience frequent staff shortages. Besides the mentioned problem, our data demonstrated that objective factors related to the donor do not have a big influence on patients outcomes. Donor liver enzymes, days in the ICU, BMI and vasoactive drugs had no negative impact on outcomes, which is in contrast with previous studies [2,31,36,37]. It is possible that the strength of recipient factors diminished the power of donor-related factors in our results.

The finding that a lower rate of NO was associated with dobutamine use in the maintenance of the donor was interesting. In our best study model (model IV), the probability of negative outcome was 46% lower in cases with donors using dobutamine. It is important to highlight the effect of this factor in association with other modeled variables [crude RR = 0.63 (95% CI: 0.43–0.92) vs. adjusted RR = 0.54 (95% CI: 0.36–0.82)]. Dobutamine is a catecholamine derivative with specificity for beta-1 adrenergic receptors and is commonly used as a cardiotonic agent after cardiac surgery and during dobutamine stress echocardiography [38]. Noradrenaline is the first line vasoactive drug, and the association with other vasoactive drugs, as dobutamine, is used when the patients or donors are managed intensively (ICUs) by doctors. In the ICUs they are better maintained integrally and so they are more prone to stay stable. By the other hand, in emergency halls donors are rarely seen by doctors, and the nurses tend only to increase noradrenaline levels when they have hypotension. The care on these places tend to be worse, donors stay hypotenses and unstable for a long period of time and rarely there are an association of vasoactive drugs. Thus, although there is no similar report in the literature, we speculate that this finding may also reflect better macrohemodynamic care. More studies are needed to understand the relationship between the dobutamine using in donors with the lower probability of failure in the recipient, therefore, for now, we can conclude that it is not a random association.

Prior to the database analysis, we performed a survey with transplant experts to evaluate the factors most significantly associated with negative outcomes following liver transplants. This analysis resulted in the theoretical framework analyzed herein (Fig 2). The results confirmed those of previous studies [2,31,37]. Curiously, some of these factors, such as cold and warm ischemia time, had no impact in this analysis. However, these factors have been suggested to be hypothetically the most important. This phenomenon likely occurred because transplant teams controlled withdrawal times, organ transport and transplant and/or mortality in this dataset. These factors are more strongly associated with the clinical status of recipients.

These experts are familiar with the specialized literature and attend international meetings regularly; however, despite their experience in the field, their knowledge of global-scale data can obscure their recognition of the realities in their own countries. The contrast between the survey and data presented herein reinforces the need for region-specific versus internationally standardized scores.

The MLG multiple model was used to assess the impact of factors on the NO rate and no survival analysis, as used in previous studies [26,39,40]. In survival analyses, the dependent variable (the outcome) is always the time to occurrence of a specific event. Thus, survival analysis compares the speed with which participants develop specific events. In contrast, the classical dependent variable statistical analysis involves the actual occurrence of a certain event (developing a disease, cure, side effects, etc.) compared to the proportions of patients who develop the event after a certain period of time [41,42]. In this paper, the patient’s follow-up is intrinsically linked with outcomes; thus, some factors that have not been identified in previous studies using survival analysis methods were shown to impact incidence density (rate) in this study. This strategy was used because some variables did not exhibit the proportional hazard required in Cox survival analysis.

The large retrospective cohort dataset was a strength in this study [43]. Additionally, analyses were performed with a statistical model that allows for multiple adjustments of effects factors and analysis of confounding effects. The hierarchical approach was consistent with biomedical knowledge in organizing the regression model. The main limitation of this study was associated with the quality of the data records in public databases, the collection of which involved records being sent to multiple transplant centers (especially data on donors) from the state secretary of health. Of course this data are from one region in one specific country, and for been considered its use in a large scale it needs to be validated in another population. However it is important to have such predictor outcome studies outside USA and Europe. The accuracy of this process is not known.

We found results lower than expected regarding patients and graft survival, but after analyzing it we could understand that they are mailing due to recipient factors. As we could see in other countries the MELD system allocation, with many teams competing with each other for not enough organs and pushing the limits for the sicker patients is harmful and bring down the outcomes. However, instead to look for another allocation system as other authors, the present study allowed a better dealing with the MELD system. We are now, in our region, trying to avoid the futile transplant. We are paying more attention on very sick patients, with high MELD score, especially in those with many organs support or after recovering from septic treatments. These measures are improving the results.

We demonstrated the greater importance of recipient disease stage vs. donor factors in predicting post-transplant results. This study leaded to a better dealing with the MELD system and it is improving the results. Future studies will be conducted with this dataset to develop a national liver transplant algorithm. We intend to use the variables that have been shown to impact on the probability of negative outcome raised in this study and intelligent computing tools to develop a model applied to the Brazilian reality.

## Conclusions

The current study confirms that both donor and recipient characteristics must be taken into consideration with respect to post-transplant outcomes and prognostic scores. Our data demonstrated that recipient health status was more important than donor status in predicting transplant outcomes. These results bring a better comprehensive of the harmful of pushing the limits of transplanting very sick patients. Moreover, it also allows us to better deal with MELD allocation system in an organs shortage reality associated with a very competitive scenario.
